# Steady State Bioequivalence of Generic and Innovator Formulations of Stavudine, Lamivudine, and Nevirapine in HIV-Infected Ugandan Adults

**DOI:** 10.1371/journal.pone.0003981

**Published:** 2008-12-19

**Authors:** Jayne Byakika-Tusiime, Leslie W. Chinn, Jessica H. Oyugi, Celestino Obua, David R. Bangsberg, Deanna L. Kroetz

**Affiliations:** 1 Division of Epidemiology, School of Public Health, University of California Berkeley, Berkeley, California, United States of America; 2 Department of Biopharmaceutical Sciences (LWC and DLK), University of California San Francisco, San Francisco, California, United States of America; 3 Infectious Diseases Institute and Makerere University Faculty of Medicine, Kampala, Uganda; 4 Department of Pharmacology and Therapeutics, Makerere University, Kampala, Uganda; 5 Massachusetts General Hospital, Harvard Medical School and Harvard Initiative for Global Health, Boston, Massachusetts, United States of America; University of Cape Town, South Africa

## Abstract

**Background:**

Generic antiretroviral therapy is the mainstay of HIV treatment in resource-limited settings, yet there is little evidence confirming the bioequivalence of generic and brand name formulations. We compared the steady-state pharmacokinetics of lamivudine, stavudine and nevirapine in HIV-infected subjects who were receiving a generic formulation (Triomune®) or the corresponding brand formulations (Epivir®, Zerit®, and Viramune®).

**Methodology/Principal Findings:**

An open-label, randomized, crossover study was carried out in 18 HIV-infected Ugandan subjects stabilized on Triomune-40. Subjects received lamivudine (150 mg), stavudine (40 mg), and nevirapine (200 mg) in either the generic or brand formulation twice a day for 30 days, before switching to the other formulation. At the end of each treatment period, blood samples were collected over 12 h for pharmacokinetic analysis. The main outcome measures were the mean AUC_0–12h_ and C_max_. Bioequivalence was defined as a geometric mean ratio between the generic and brand name within the 90% confidence interval of 0.8–1.25. The geometric mean ratios and the 90% confidence intervals were: stavudine C_max_, 1.3 (0.99–1.71) and AUC_0–12h_, 1.1 (0.87–1.38); lamivudine C_max_, 0.8 (0.63–0.98) and AUC_0–12h_, 0.8 (0.65–0.99); and nevirapine C_max_, 1.1 (0.95–1.23) and AUC_0–12h_, 1.1 (0.95–1.31). The generic formulation was not statistically bioequivalent to the brand formulations during steady state, although exposures were comparable. A mixed random effects model identified about 50% intersubject variability in the pharmacokinetic parameters.

**Conclusions/Significant Findings:**

These findings provide support for the use of Triomune in resource-limited settings, although identification of the sources of intersubject variability in these populations is critical.

## Introduction

Generic drugs provide patients with lower-cost alternatives to the more costly brand name drugs. Production of more affordable generic antiretroviral medications (ARVs) has greatly boosted global efforts to scale up access to these life-saving medications. Today, more than half of all ARV prescriptions in sub-Saharan Africa are filled with generic drugs. The use of generics has resulted in substantial savings to consumers and governments. These drugs have dramatically reduced the morbidity and mortality due to HIV/AIDS, and drug quality is a key factor in attaining the long term goals of sustained viral suppression with minimal drug resistance. Triomune®, a fixed-dose generic combination of lamivudine, stavudine and nevirapine, has been shown to be bioequivalent to its innovator counterparts (Epivir®, Zerit® and Viramune®) in a single dose study in healthy Indian volunteers [1]. However, drug pharmacokinetics and pharmacodynamics may vary in ethnically distinct populations of HIV-infected patients. Furthermore, since this is chronic therapy, there is a need to evaluate the steady-state pharmacokinetics of these drugs in the target population. A study conducted in Malawian HIV-infected adults showed that Triomune was not strictly bioequivalent to its innovator cousins at steady state [2], but this has not been replicated in other populations. Assurance of continual exposure to optimal plasma concentrations of these medications is essential to avoid the development of drug resistance resulting from sub therapeutic plasma concentrations. The goal of this study was to compare the pharmacokinetics of Triomune and its innovator counterparts in HIV-infected Ugandans with advanced disease. In addition, we sought to investigate the source of variation in pharmacokinetic parameters.

## Materials and Methods

### Ethics committee review and informed consent

The study protocol and informed consent forms were reviewed and approved by the Makerere University Faculty of Medicine Ethics Committee and the University of California San Francisco Committee on Human Research. All subjects provided written informed consent prior to participation in the study.

### Study setting and subject selection

Subjects were recruited from an ongoing cohort study (Adherence Monitoring Uganda (AMU)) in Kampala, Uganda. AMU was an observational study of adherence and treatment response among individuals on self-pay HIV generic antiretroviral therapy conducted from 2002–2007 [3], [4]. AMU cohort members with a body weight of 60 kg or greater were approached by a member of the research team and invited to participate in the bioequivalence study; those who were interested and fulfilled other selection criteria were enrolled into the study. Subjects received quarterly adherence assessments using unannounced home pill count, Medication Event Monitoring System (MEMS) and laboratory monitoring of CD4 T cells, HIV RNA, and hematological function. All subjects had been taking Triomune-40 (stavudine 40 mg, lamivudine 150 mg, nevirapine 200 mg) twice daily for at least three years. Most of the subjects attended the Infectious Diseases Clinic in Mulago, Uganda. Mulago Hospital is the main teaching and referral hospital in Uganda and the Infectious Diseases Clinic is a specialized HIV/AIDS clinic that treats over 13,000 patients, ∼5000 of whom receive antiretroviral therapy.

Participants received a medical and laboratory examination no less than seven days prior to enrollment in this pharmacokinetic sub-study to exclude active opportunistic infections. A urine pregnancy test was performed on all women participants. Subjects were excluded if they had active tuberculosis, were taking drugs known to interefere with the metabolism or transport of the study drugs (rifampicin, cimitedine, erthyromycin, ketoconazole, carbamazepine, phenobarbitone and phenytoin), had gastrointestinal problems, hepatitis, hemoglobin less than 7 mmol/L for men and 6.5 mmol/L for women, liver and renal function test results 5 or 1.5 times the upper limit of normal, respectively, or were pregnant. We also excluded those patients who expected to change their regimen or move out of the study area within two months. Subjects on daily cotrimoxazole (trimethoprim/sulfamethoxazole) prophylaxis were included.

### Study design

This study utilized an open-label, randomized, crossover design comparing the pharmacokinetics of generic and trade formulations of stavudine, lamivudine and nevirapine under fed conditions. Subjects were randomized to one of two formulations. Formulation 1 (generic) was a single tablet containing lamivudine (150 mg), stavudine (40 mg) and nevirapine (200 mg) (Triomune-40). Formulation 2 (brand) was a single tablet of lamivudine (150 mg, Epivir) plus one 40 mg tablet of stavudine (Zerit) and one 200 mg tablet of nevirapine (Viramune). Each formulation was taken twice daily. Seven subjects were randomized to the generic-to-brand arm and 11 were randomized to the brand-to-generic arm. Subjects took each formulation for 30 days prior to pharmacokinetic sampling. The generic formulation (Triomune-40) was manufactured by Cipla (Mumbai, India) and the brand formulations by: Zerit, Bristol- Myers Squibb, Princeton, NJ, USA; Epivir, GlaxoSmithKline, Middlesex, United Kingdom and Viramune, Boehringer Ingelheim, Ingelheim am Rhein, Germany. The study was conducted between March and May, 2006.

### Study procedures

To monitor adherence, study drugs were dispensed to the participants in a bottle with an electronic cap that recorded each pill bottle opening using the Medication Event Monitoring System (MEMS) (Aardex. (2004), Union City, CA). In addition, the pills were counted and the number recorded manually at each visit. Adherence was also assessed by a 3-day self report and 30-day visual analogue scale [3].

Participants were admitted to the hospital ward the night before the pharmacokinetic sampling and administration of the 8 p.m. evening dose was witnessed by research staff. Participants were fasted overnight and were instructed not to take their morning dose(s) or eat until the first blood sample had been drawn the next day.

Each subject had an indwelling catheter inserted into an arm vein for drawing serial blood samples. A 6 ml blood sample was obtained before antiretroviral medications were administered (t = 0 hr) during a witnessed dose at 8 a.m. Additional 6 ml blood samples were obtained at 0.5, 1, 1.5, 2, 3, 4, 6, 10 and 12 hr post-dosing. Food and water intake were controlled during the study. After the 12 hr sample collection participants were given the alternate formulation in a MEMS bottle and asked to report back to the clinic after 30 days. At the next visit, identical procedures were repeated.

Blood samples were collected in vacutainer tubes containing EDTA as the anticoagulant. Blood samples were immediately delivered to the Makerere University Johns Hopkins University Collaboration (MUJHU) laboratory where plasma was separated by centrifugation at 900*g* for 10 min and then stored at −70°C until analysis. Samples were transported in a single batch on dry ice to the Department of Biopharmaceutical Sciences at the University of California San Francisco for analysis.

### Analysis of plasma samples

Prior to drug extraction, plasma samples (including controls) were heated at 56°C for 90 min to inactivate virus. Heat treatment was determined to have no quantifiable effect on drug stability or concentration. Drug concentrations were analyzed using a method reported previously [5]. Briefly, stavudine, lamivudine, and nevirapine were extracted simultaneously from 0.5 ml of plasma using solid-phase extraction columns (Oasis HLB Extraction Cartridges, Waters Corporation, Milford, Massachusetts, USA) and eluted with 1 ml of mobile phase, consisting of 0.1% glacial acetic acid in acetonitrile∶water (80∶20, v/v). Metaxalone was used as an internal standard. Samples were subjected to LC/MS/MS on an API 4000 system (Applied Biosystems, Foster City, California, USA) using a Waters 717plus autosampler and a Symmetry C18 (150 mm×3.9 mm i.d., 5 µm particle size, Waters Corporation) analytical column. The flow rate was 0.4 ml/min. All peaks were quantified using multiple reaction monitoring (MRM) mode to study the conversions from parent to product ion (m/z), and data were collected using Analyst version 1.4 (Applied Biosystems). The precision of the assay, as measured by the interassay coefficient of variation of control samples, was <13.2%, <15.2%, and <14.8% for stavudine, lamivudine, and nevirapine, respectively.

### Pharmacokinetic analysis

Pharmacokinetic parameters were determined from plasma concentrations based on a non-compartmental model with extravascular input (Model 200) using WinNonLin software, version 5.2 (Pharsight Corporation, Mountain View, CA, USA). The AUC was calculated using the log-linear trapezoidal rule, in which the linear trapezoidal rule was used up to C_max_, and thereafter the logarithmic trapezoidal rule was used. C_max_ and t_max_ were directly observed from the concentration-time data. Geometric mean ratios (GMR) and 90% confidence intervals (CI) for AUC_0–12h_ and C_max_ were used in the determination of bioequivalence, as defined by a 90% CI range of 0.80–1.25.

### Statistical Analyses

A sample size of 18 subjects was estimated using a formula by Zhang *et al.* [6] to provide 80% power to detect approximately a 20% difference on a log scale in AUC_0–12h_ and C_max_ between the brand formulation and the generic formulation. Power was based on findings from an earlier study [1] in which the coefficients of variability (CV) for stavudine, lamivudine and nevirapine AUC were 16%, 18% and 21%, respectively.

Primary parameters for statistical analyses were AUC_0–12h_ and C_max_ of lamivudine, stavudine and nevirapine and their intra- and intersubject variances. Differences between treatments with respect to AUC_0–12h_, C_max_ and intra- and intersubject variances were assessed using the mixed random effects model. In particular, we used the simple random intercept model with bootstrapped standard errors because the drug concentrations, even after log transformation, were not normally distributed. The model employed for this study included sequence, period, and treatment as fixed effects and subjects (nested within sequence) as the random effects. The primary analysis was conducted on log-transformed (base e) AUC_0–12h_ and C_max_. Measured values of C_0h_ and C_12h_ were compared to assess the presence of steady-state conditions and to evaluate adherence.

## Results

### Subjects

Twenty HIV-positive adults (8 males, 12 females) were enrolled in the study. Two subjects were excluded from the analysis because their plasma samples were not sufficient for pharmacokinetic analysis. The analysis therefore includes 18 participants. Participants ranged from 28 to 50 years of age (mean±SD; 37.4±6.0), weighed 68.3±6.6 kg, averaged 165±9.4 cm in height, and had a mean body mass index of 25.1±3.4 kg/m^2^. Patient characteristics did not differ between randomization arms (Table 1). All participants had been on the generic lamivudine/stavudine/nevirapine formulation for at least 36 months. Ten participants were taking prophylactic cotrimoxazole during the study. All subjects were physically healthy based on their medical examination and results from clinical laboratory tests. No tuberculosis, malabsorption, nausea, emesis, abdominal discomfort, chronic diarrhea, or hepatitis was reported. All females had a negative pregnancy screen.

**Table 1:** Baseline characteristics of participants according to randomization arm.

	Generic→Brand	Brand→Generic
Number	7	11
Mean age [years(SD)]	35.3 (5.3)	38.8 (6.3)
Sex (M/F)	3/4	4/7
Mean weight [kg (SD)]	68.4 (10.2)	68.2 (3.8)
Mean height [cm (SD)]	163 (8.7)	167 (9.8)
Mean body mass index [kg/m^2^ (SD)]	26.0 (4.4)	24.5 (2.8)
Mean HIV RNA [log_10_ copies/ml (SD)]	2.8 (0.6)	2.8 (0.5)
HIV RNA<400 copies/ml [n (%)]	5 (71.4)	10 (90.9)
Mean CD4 T cell count [cells/µl (SD)]	319 (116)	402 (267)
Mean Hemoglobin [mmol/l (SD)]	14.7 (1.5)	13.8 (1.9)
Mean alanine aminotransferase [U/L (SD)]	26.7 (7.34)	30.8 (18.2)
Mean aspartate aminotransferase [U/L (SD)]	27.7 (8.9)	29.9 (8.54)
Mean serum creatinine [mg/dL (SD)]	1.1 (0.1)	0.9 (0.2)
On prophylactic cotrimoxazole [n (%)]	5 (71.4)	5 (45.5)

### Adherence and steady-state drug concentration

Based on MEMS adherence records, mean adherence for the 30 days prior to the first and second pharmacokinetic study visits was 99.7% and 99.0%, respectively. Fifteen out of eighteen subjects had 100% adherence before the first pharmacokinetic sampling and sixteen out of eighteen subjects had 100% adherence before the second pharmacokinetic sampling. Each of the three subjects with incomplete adherence before the first pharmacokinetic sampling had 98% adherence and of the two subjects with incomplete adherence before the second pharmacokinetic sampling, one had 95% while the other had 88% adherence. In addition to the MEMS and pill count we also did a three day self report on the morning of the pharmacokinetic sampling asking about adherence over the previous three days. For each of these participants, three day self reported adherence prior to pharmacokinetic sampling was 100% and the MEMS report showed that all doses had been taken in the week preceding pharmacokinetic sampling for two subjects. For the subject with 88% adherence at the second pharmacokinetic draw, the MEMS report showed that he had taken only 9 out of 14 prescribed doses. For the remaining two subjects with incomplete adherence [95% and 98%] non-adherence did not occur immediately prior to pharmacokinetic sampling.

Trough plasma concentrations in each subject prior to the study dose of medication for each period were determined to confirm steady state conditions. All patients had measurable levels of each drug prior to administration of both the generic and brand formulations. Mean trough plasma concentrations were similar for generic and brand lamivudine and stavudine, but trough nevirapine concentrations were significantly higher with the generic formulation during period 2 (Table 2).

**Table 2:** Trough plasma concentrations prior to initiation of pharmacokinetic study.

	Plasma Concentration (ng/ml)^a^		P-value
	Brand	Generic	
Period 1			
3TC	303±270	212±160	0.43
D4T	195±342	161±245	0.86
NVP	8230±4270	6160±1890	0.25
Period 2			
3TC	468±486	391±405	0.72
D4T	283±331	511±547	0.44
NVP	4770±952	9300±3640	0.01

a Plasma concentrations are expressed as mean±SD.

### Bioequivalence Evaluation

**Figure 1:**
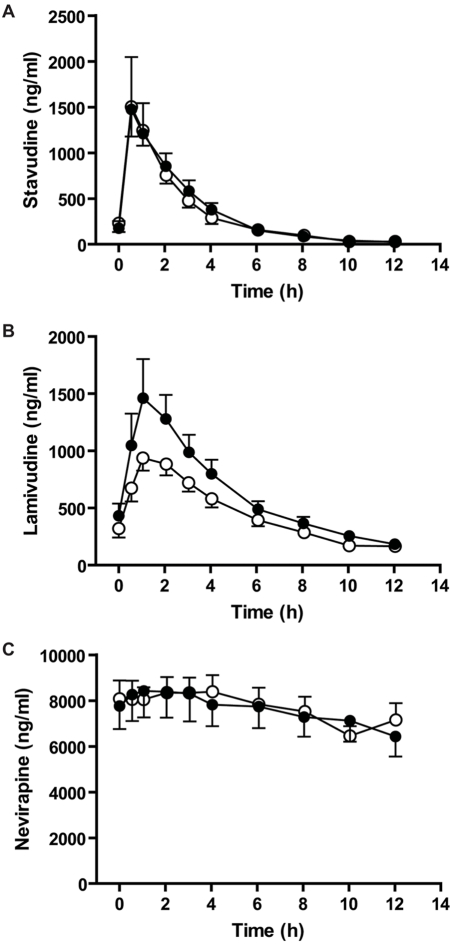
Plasma concentration-time profiles for brand and generic stavudine, lamivudine and nevirapine. Mean plasma concentration-time profiles of (A) stavudine, (B) lamivudine and (C) nevirapine in 18 subjects after oral administration of brand (closed symbol) or generic (open symbol) formulation. Each value represents the arithmetic mean±SE for 18 subjects in each arm.

The mean concentration-time profiles over 12 hours for each drug after administration of the generic and brand formulations are shown in Figure 1. There is little noticeable variation in plasma levels of stavudine or nevirapine between the brand and generic formulations. In contrast, lamivudine levels are much higher following the dosing of the brand name formulation compared to the generic. As expected based on its long half-life, nevirapine levels were relatively constant during the 12 hr study period.

Both AUC_0–12h_ and C_max_ were analyzed as bioequivalence markers. The geometric mean ratios for AUC_0–12h_ were close to unity for all three compounds, suggesting similar exposure with the two formulations. The largest variation was seen for lamivudine, with a 20% reduction in exposure with the generic formulation. The FDA definition for bioequivalence of a 90% confidence interval of the geometric mean ratio between 0.8 and 1.25 is not met for AUC_0–12h_ for any of these drugs. Similar results were found for C_max_, with nevirapine being the only drug that meets the bioequivalence criteria (GMR = 1.1, 90% CI 0.95–1.23) (Table 3). For lamivudine, the C_max_ was 20% lower with the generic compared to the brand formulation while for stavudine the C_max_ was 30% higher with the generic compared to the brand formulation. Because the concentration-time profiles (Fig. 1) are drawn on an ordinary scale the difference in C_max_ for stavudine is not very noticeable given that the arithmetic means for the brand and generic were very close.

**Table 3:** Pharmacokinetic parameters of generic and brand name nevirapine, stavudine and lamivudine.

Pharmacokinetic Parameter^a^	Arithmetic Mean^b^		Geometric Mean^b^		GMR^c^
	Generic	Brand	Generic	Brand	
**Nevirapine**					
C_max_ (mg/L)	9.2±3.1	9.6±5.5	8.8±3.1	8.4±5.5	1.1 (0.95–1.23)
AUC_0–12h_ (h*mg/L)	91.5±35.2	88.2±45.7	85.8±35.2	79.2±45.7	1.1 (0.95–1.31)
t_max_ (h)	1.8±1.8	2.1±2.6			
**Lamivudine**					
C_max_ (mg/L)	1.1±0.5	1.7±1.4	1.0±0.5	1.3±1.4	0.8 (0.63–0.98)
AUC_0–12h_ (h*mg/L)	5.6±2.5	7.5±4.9	5.2±2.5	6.4±4.9	0.8 (0.65–0.99)
t_max_ (h)	1.1±0.8	1.2±0.6			
**Stavudine**					
C_max_ (mg/L)	1.9±1.1	1.8±2.0	1.6±1.1	1.3±2.0	1.3 (0.99–1.71)
AUC_0–12h_ (h*mg/L)	4.1±2.4	4.2±3.9	3.6±2.4	3.4±3.9	1.1 (0.87–1.38)
t_max_ (h)	08±0.5	1.1±0.8			

a Abbreviations used are C_max_, maximum plasma concentration, AUC, area under the concentration-time curve, and t_max_, time to maximum plasma concentration.

b Pharmacokinetic parameters are given as the mean±SD.

c GMR, geometric mean ratio; the 90% confidence interval is given in parentheses.

A mixed random effects model demonstrated significant sequence effects for both nevirapine log transformed C_max_ (p<0.0001) and AUC (p<0.0001) as outcomes. Intersubject variability for log transformed C_max_ ranged from 0.24–0.40 and that for AUC ranged from 0.23–0.42. Intrasubject variability for C_max_ ranged from 0.21–0.44 and AUC ranged from 0.25–0.36 (Table 4). Intersubject variability accounts for approximately half of the variability in the C_max_ and AUC estimates.

**Table 4:** Inter- and intrasubject variability in log transformed pharmacokinetic parameters.

	Intersubject Variability^a^	Intrasubject Variability^a^	Correlation Coefficient (ρ)^b^
**logC_max_**			
Lamivudine	0.37	0.34	0.53
Stavudine	0.40	0.44	0.46
Nevirapine	0.24	0.21	0.57
**logAUC_0–12_**			
Lamivudine	0.33	0.33	0.50
Stavudine	0.42	0.36	0.57
Nevirapine	0.23	0.25	0.46

a Inter- and intrasubject variability indicate the standard deviation of the pharmacokinetic parameters between or within subjects, respectively.

b ρ is the proportion of the total variability that is due to the variability between subjects.

## Discussion

We found generic stavudine, lamivudine and nevirapine in the form of Triomune fails to meet strict bioequivalence criteria in patients with objectively confirmed adherence on stable therapy. While Triomune failed to meet strict bioequivalence, the differences were relatively minor and are unlikely to be clinically significant. Our results are similar to a recently reported bioequivalence study carried out in Malawian HIV-infected patients [2]. One exception is that in the former study, the generic Triomune formulation resulted in a significant increase in stavudine C_max_ compared to the brand name. In both the present study and in the Malawian report, plasma levels of nevirapine following administration of either the generic or brand formulations were higher than those previously reported in Caucasian HIV patients [2], [7]. A large fraction of nevirapine is metabolized by the polymorphic CYP2B6 enzyme [8], [9]. Whether the elevated levels of nevirapine in these African populations is related to the higher allele frequency of the reduced function *CYP2B6* 516G>T polymorphism in these populations should be investigated in larger samples.

There are several reasons why the differences in drug exposure between brand and generic medication are unlikely to be clinically significant. Over 70% achieved undetectable viral load levels (<400 copies/ml) and had significantly improved their CD4 count at 12 and 24 weeks [3], [4]. Even in the case of lamivudine, for which C_max_ and AUC were decreased 20–30% with the generic formulation compared to brand name, the plasma concentrations were similar to those reported following a single dose of Triomune or a second combination drug containing abacavir, lamivudine and zidovudine to healthy subjects [1], [10]. Furthermore, the mixed random effects model found no statistically significant difference in C_max_ or AUC between the formulation types for any of the three drugs (results not shown).

We found a high degree of variability between study subjects. The random effects model produced correlation coefficients of about 50% for both log transformed C_max_ and AUC values (Table 4). This implies that about 50% of all variability in these parameters was due to differences between study subjects. The interindividual variability in the pharmacokinetics of stavudine, lamivudine, and nevirapine may be attributable to various sources, such as environmental or genetic factors, that were not addressed in this study. In particular, genetic polymorphisms in transporters or drug-metabolizing enzymes for which these drugs are substrates may affect the pharmacokinetics of these drugs. For example, the cytochrome P450 2B6 (CYP2B6) enzyme, which is involved in nevirapine metabolism, contains a genetic polymorphism (516G>T) that has been shown to substantially decrease hepatic protein expression and function [11]. In patients with HIV, this polymorphism has been significantly associated with increased nevirapine plasma levels [12]. While the nucleoside reverse transcriptase inhibitors lamivudine and stavudine are not extensively metabolized in the liver, it is possible that polymorphisms in membrane transporters could influence the bioavailability of lamivudine and stavudine, thereby modulating plasma drug levels. Recently, a polymorphism in the MRP4 transporter was associated with intralymphocytic lamivudine levels in HIV patients [13]; conceivably, this polymorphism may similarly affect MRP4 activity in enterocytes, where drug absorption occurs. Due to the relatively small minor allele frequencies of these polymorphisms, a larger study population is needed to address these pharmacogenetic questions.

We observed a significant difference in the mean nevirapine trough concentrations between the brand and the generic formulation. However, these steady state plasma nevirapine concentrations are far above the concentration required to inhibit 50% viral replication *in vitro*; the IC50 for nevirapine is 10.6 ng/ml [14] so this difference may not have clinical relevance. The first round of pharmacokinetic sampling does not show a significant difference in the mean nevirapine trough concentrations between the brand and generic formulation. This implies that the observed difference is not a systematic difference in the trough concentrations of the two formulations and may be due to the observed sequence effects. A limitation of our study was that we did not conduct drug content assays and in vitro dissolution tests. A drug content assay for each formulation would rule out formulation problems. In vitro dissolution testing would reveal variations in drug degradation between brand and generic formulations that could lead to *in vivo* differences in the rate of nevirapine absorption.

Our analysis model identified significant sequence effects for nevirapine C_max_ and AUC. Although the exact causes of the sequence effects are not known, ‘real’ differences between the groups could contribute to the observed effects. While our study groups were similar in most baseline characteristics, the brand to generic group was generally healthier than the generic to brand group. Ninety one percent (10/11) of the brand to generic group was virologically suppressed (<400 copies/ml) compared to 71% (5/7) in the generic to brand group (Table 1). In addition, mean CD4 cell count in the brand to generic group was about 80 units more than the mean CD4 count in the generic to barnd group (402 cells/µl vs. 319 cells/µl). To control for this empirical confounding, baseline CD4 cell count and viral load were added to the model. We found a significant association between baseline CD4 cell count and nevirapine C_max_ (p = 0.007) and AUC (p = 0.02). Higher CD4 cell count was associated with lower drug levels. This difference in health status may explain the observed sequence effects.

Triomune-40® contains a higher dose of stavudine than is currently recommended in treatment guidelines. Current WHO guidelines now recommend a 12 hourly dose of 30 mg for all patients, irrespective of weight [15]. This limits applicability of our results to patients currently on antiretroviral therapy. Studies exploring bioequivalence of fixed dose combination formulations containing 30 mg of stavudine are needed.

In summary, the steady-state pharmacokinetics of the generic formulation (Triomune-40®) in HIV-infected patients did not meet the strict bioequivalence requirement set by the FDA when compared to the brand name formulations of lamivudine, stavudine, and nevirapine. However, based on the measured plasma levels, the generic formulation is expected to produce a similar therapeutic response as the brand name formulations. There was a large degree of interindividual variability in antiretroviral exposure. Variability could have been due to individual disease state and progression, or the genetic variation within the subjects. Understanding the exact sources of this variability will be important for optimization of therapy. These results suggest that bioequivalence and pharmacokinetic studies are needed in specific populations in which these medications are used, to account for unique characteristics that may influence drug disposition. Drug regulatory bodies in countries in which generic antiretroviral medications are used should endeavor to test all antiretrovirals imported into the country to ensure drug quality.
